# Prevalence of emotional symptoms in Chilean oncology patients before the start of chemotherapy: potential of the distress thermometer as an ultra-brief screening instrument

**DOI:** 10.3332/ecancer.2014.437

**Published:** 2014-06-16

**Authors:** Jorge Calderón, Cristóbal Campla, Nicole D’Aguzan, Soledad Barraza, Oslando Padilla, Cesar Sánchez, Silvia Palma, Matías González

**Affiliations:** 1 Psychiatry Department, Pontificia Universidad Católica de Chile, Santiago 8330024, Chile; 2 Cancer Center, Pontificia Universidad Católica de Chile, Santiago 8330024, Chile; 3 Department of Public Health, Pontificia Universidad Católica de Chile, Santiago 8330024, Chile

**Keywords:** cancer, emotional stress, depression, screening

## Abstract

Emotional distress (ED) is greater for oncology patients in comparison with the general population, and this has implications for the quality of life of the patient and his/her family, adherence to the treatment, and eventually, survivorship. In general, the detection of these symptoms is low, which explains the need for detection systems appropriate to the clinical reality of the oncology team. The objective of this study is to evaluate for the first time the usefulness of an ultra-brief screening instrument [distress thermometer (DT)], in a group of Chilean oncology patients. A total of 166 outpatients were evaluated at the Cancer Center of the Pontificia Universidad Católica de Chile, before starting chemotherapy. Two screening instruments were applied: Hospital Anxiety and Depression Scale (HADS) and DT. The application of HADS resulted in a prevalence of 32.7% of anxiety symptoms (HADS-A ≥ 8), 15.7% of depression symptoms (HADS-D ≥ 8), and 39.8% had a total score of HADS-T ≥ 11. The DT resulted in the prevalence of 32.5% of distress or ED (DT ≥ 5). The validity of the DT was evaluated as a screening tool in comparison with HADS, observing, in relation to the anxiety scale (HADS-A), a sensitivity of 88.9% and specificity of 78.4% (DT ≥ 4); depression (HADS-D), a sensitivity of 69.2% and specificity of 74.3% (DT ≥ 5); and in relation to the total scale (HADS-T), a sensitivity of 68.2% and specificity of 73.0% (DT ≥ 4). This study demonstrates the elevated prevalence of emotional symptoms in Chilean oncology patients, before the start of chemotherapy, and confirms the potential of the DT as a brief screening instrument with easy application. The DT will allow the clinician to increase the detection threshold in the Chilean oncology population, intervene in a timely manner, and contribute to the comprehensive handling of the oncology patient without affecting the time needed for assistance.

## Introduction

In 2012, around 14.1 million new cancer cases were diagnosed worldwide, reaching a mortality rate of around 8.2 million. The largest cancer incidence around the world was of lung cancer, with 1.8 million new cases, followed by breast cancer, with 1.67 million [1].

In Chile, cancer is the second cause of death after cardiovascular illnesses, causing in 2005 24.8% of total deaths and reaching, in 2008 a figure of 21,824 deaths, with a rate of 130 deaths for every 100,000 inhabitants [2].

The prevalence of emotional distress (ED) in oncology patients is of about 35% in the course of the illness [3, 4], and it is greater for young people, depending on the location of the tumour (greater risk for cerebral tumours) and the oncology illness of worst prognosis, with a greater risk for patients with a survival prognosis of less than one year [5].

Psychiatric co-morbidity in cancer patients increases the number of days of hospitalisation, the demand for medical attention, and the risk of suicide [6, 7]; it delays adaptation to the cancer diagnosis for at least a month, and it is associated with lower adherence to anti-neoplastic treatments [8]. Depression symptoms increase sensitivity to pain and affect the rank and intensity of the side effects of the treatment, with a negative impact on the physical well-being and the social functioning of the patient [9].

As for the neoplastic illness prognosis, a meta-analysis of prospective studies found a statistically significant, although small, connection between depression symptoms and mortality, with an unadjusted relative risk of 1.25 (IC 95% 1.12–1.40; *p* < 0.001) increasing to 1.39 when given a major depressive disorder diagnosis (IC 95% 1.10–1.89; *p* = 0.03) [10].

In Chile, there is no specific record of psychiatric morbidity in oncology patients, and standardised instruments to study these disorders in said population have not been evaluated. The use of the Hospital Anxiety and Depression Scale (HADS) in a study of patients with chronic pathologies in an advanced stage (of which 77.6% were oncology patients) reported anxiety and depression symptoms in 51.1% and 27.9% of the patients, respectively (cut-off point ≥ 8). Considering only the ‘clinically relevant’ cases (cut-off point ≥ 11) in both sub-scales, the frequency was of 30.2% and 11.6%, respectively [11].

There are several instruments used to evaluate the psychosocial health of people with cancer, and although these instruments have been used mainly in research, there is a growing interest in incorporating them into clinical practice as part of the standardised evaluation. Amongst these are highlighted the HADS, Psychological Distress Inventory, Brief Symptom Inventory, and others. Although many patients who complain of emotional problems do not meet the criteria for major depressive disorder according to the DSM-IV, the presence of ED affects the experience of the patient and his/her family. This has led to the development of the concept of distress (hereafter, ED), which has even been suggested to be the ‘sixth vital sign’ and which has allowed for the formulation of ultra-brief screening tools such as the distress thermometer (DT) [9].

The aim of this study is to measure the prevalence of depression and anxiety symptoms in an outpatient group of oncology patients in a university clinical hospital in Chile, before the start of chemotherapy, and to show the usefulness and diagnostic precision of the DT in comparison with the HADS, as an ultra-brief screening tool to detect the ED in this sample. This will allow for the establishment of the psychometric properties of the DT in a group of Chilean patients and the comparison with the properties in other populations.

## Materials and methods

The study included a total of 166 consecutive patients from the Cancer Center of the Pontificia Universidad Católica de Chile. The evaluation took place immediately after the introduction talk, prior to the start of chemotherapy. The study included patients with different cancer types who will undergo chemotherapy.

The HADS was initially designed to evaluate the psychological status of patients who are physically ill, and it was used to determine the presence of depression and anxiety symptoms [12]. It has been widely accepted as an effective tool in the study of anxiety and depression symptoms in oncology patients [13–15], and it is validated in Spanish [16]. It consists of 14 questions, divided into seven questions that identify anxiety symptoms (HADS-A) and seven questions that identify depression symptoms (HADS-D). Each answer gets between 0 and 3 points, for a total of 21 points for each sub-scale and 42 for the whole scale. The greater the score, the greater the degree of anxiety or depression. In the original report, the cut-off point was 8 for suspicious cases and 11 for definitive cases, both for anxiety (HADS-A) and depression (HADS-D) scales. Our study used a value of ≥ 8 as a cut-off point in the depression and anxiety sub-scales and a value of ≥ 11 in the total scale.

The second tool used was the DT. To study ED in oncology patients in a quick, simple, and non-stigmatising manner, Roth *et al*. [17] designed the DT. Similar to the analogue visual pain evaluation scale, the patient is asked questions related to his/her ED grade in the last week on a scale of 0–10. This scale has been incorporated in the clinical practice guides for ED management of the National Comprehensive Cancer Network (NCCN) [18]. The NCCN later developed the ‘list of problems’ (LP), consisting of 34 problems, commonly experimented by oncology patients, grouped in five categories: practical, physical, family related, emotional, and spiritual. Initially, the NCCN recommended a cut-off point of 5 in the DT to determine a significant ED, which requires referral to the appropriate service. The DT together with the LP have been proven to be effective screening tools for detecting ED in patients with different types of cancer [19–21].

## Statistical analysis

Measurements for age and the different scales were calculated. The prevalence of ED amongst men and women or according to the type of cancer diagnosis was compared using the chi-square test, or the Fisher exact probability test. Receiver operating characteristic (ROC) curve analysis was performed. The level of statistical significance was set at 0.05. The analyses were made using the program SPSS Statistics for Windows, Version 17.0 (Chicago, 2008, SPSS Inc.).

## Results

A total of 166 patients were included: 109 women (65.6%) and 57 men (34.5%) between 16 and 79 years of age. The most frequent diagnoses were breast cancer (27.9%) and colon and rectum cancer (19.9%). Of the total, 78 (47.3%) patients presented curable neoplasms, 67 (40.6%) incurable, and in 21 cases (12.7%), the prognosis was uncertain (Table 1).

### Prevalence of anxiety, depression, and ED symptoms in oncology patients

There was a prevalence of anxiety symptoms (anxiety, hereafter) of 32.7% (HADS-A ≥ 8), of 15.7% for symptoms of depression (depression, hereafter; HADS-D ≥ 8) and 39.8% for the total score (HADS-T ≥ 11). The average score for HADS-A was 6.42, with a standard deviation of 3.87: in HADS-D, it was 3.69, with a standard deviation of 3.34, and in HADS-T, it was 10.34, with a standard deviation 6.57. The DT resulted in a prevalence of ED of 32.5% (DT ≥ 5), with an average score of 3.52 and a standard deviation of 2.57. The LP identified 53.6% of patients who admit having practical problems, 22.3% family problems, 80.1% emotional problems, 25.9% spiritual problems, and 88% physical problems (Table 2).

With respect to gender differences, these were not significant for anxiety (*p* = 0.12), depression (*p* = 0.23), and HADS-T (*p* = 0.30; Table 2). Neither were there significant gender differences in the problems reported, where only the spiritual problem report showed a tendency to statistical significance, with a greater amount reported in women (*p* = 0.08).

With respect to the ED measured by the DT, women showed a prevalence of 38.5% (DT ≥ 5), considerably greater than the 21.4% of men (*p* = 0.03; Table 2).

With respect to cancer type, no significant differences were found between breast cancer and the rest of the neoplasms, both in anxiety prevalence (*p* = 0.31), depression (*p* = 0.84), HADS-T (*p* = 0.83), and ED (*p* = 0.95). The same analyses took place for colorectal cancer, where considerable differences were also not found in comparison with the rest of the neoplasms both for anxiety (*p* = 0.20), depression (*p* = 0.63), HADS total (*p* = 0.34), and ED (*p* = 0.15). The analysis of these diagnoses was favoured, as they had the most prevalence. There were no significant differences in these measurements between patients with curable and incurable cancer.

### Determination of cut-off points, sensitivity, and specificity of the DT with relation to HADS

**Figure 1.**
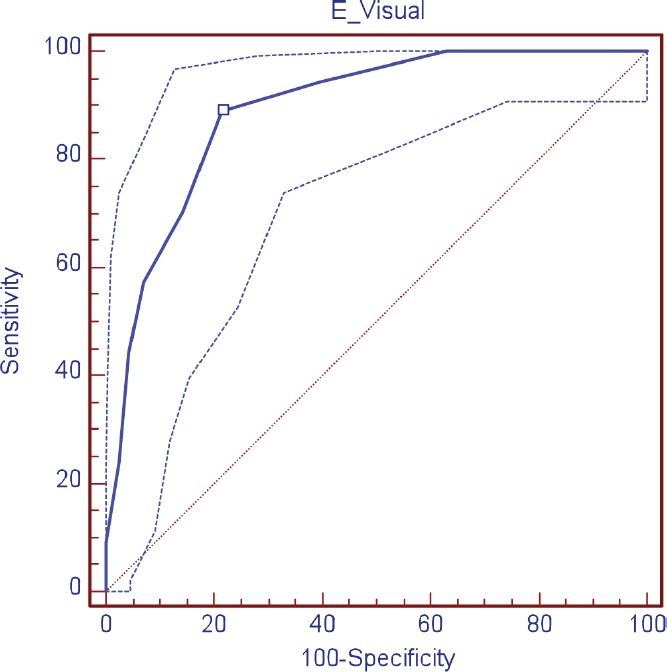
ROC curve visual scale (DT) for anxiety.

**Figure 2.**
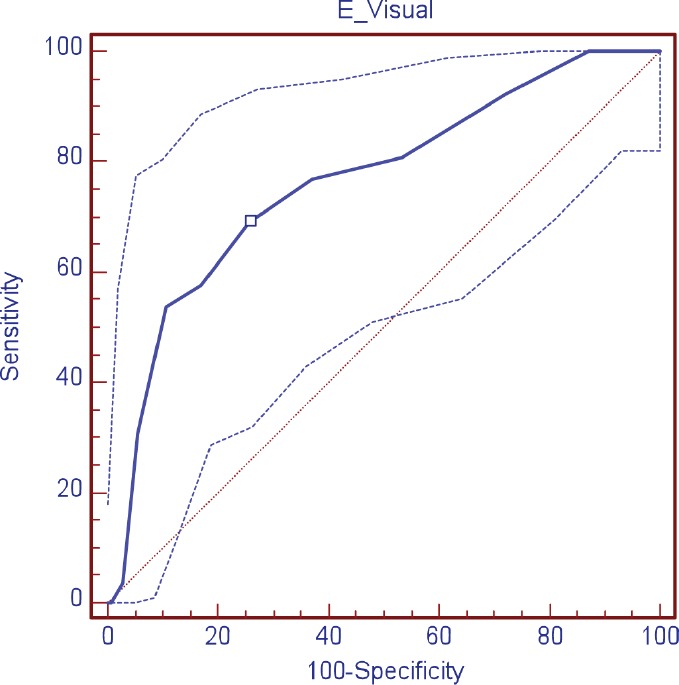
ROC curve visual scale (DT) for depression.

**Figure 3.**
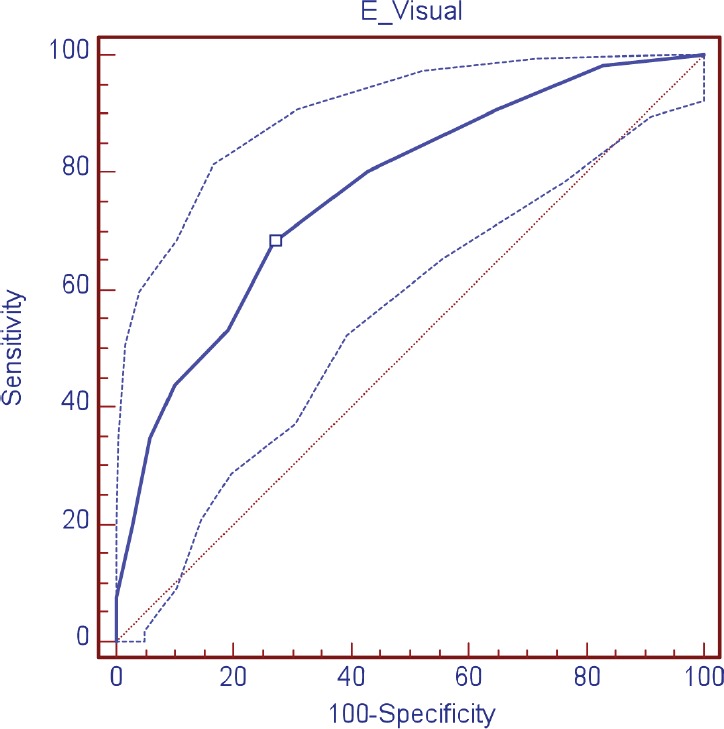
ROC curve visual scale (DT) for total HADS.

The validity of the DT was evaluated as a screening tool in comparison with HADS, observing, in relation to the anxiety scale (HADS-A), a sensitivity of 88.9% and specificity of 78.4%; area under the curve (AUC) 0.89 (DT ≥ 4); depression (HADS-D), a sensitivity of 69.2% and specificity of 74.3% AUC 0.76 (DT ≥ 5); and in relation to the HADS-T, a sensitivity of 68.2% and specificity of 73.0% AUC 0.77 (DT ≥ 4) (Table 3; Figures 1–3)

When evaluating the converging validity of the DT as a screening tool, the sensitivity and specificity of different cut-off points of the DT in relation to the HADS through the AUC curve analysis for two categories depending to the prognosis (curable or incurable) were considered. The AUC was used to calculate the precision of the cut-off points, with a range of 1 (perfect precision) to 0.5 (poor precision).

In patients with a curable prognosis, a cut-off point of DT ≥ 4 was identified for anxiety risk, a value that presented an AUC of 0.93 (IC 95% 0.86–0.98, *p* < 0.0001), sensitivity of 93.1% (IC 95% 77.2–99.0), and specificity of 87.8% (IC 95% 75.2–95.3), with a positive predictive value (VPP) of 81.8 and a negative predictive value (VPN) of 95.6 (Table 4). In patients with an incurable prognosis, the cut-off point of DT for anxiety risk was also ≥ 4, with AUC of 0.86 (IC 95% 0.8–0.9, *p* < 0.0001), sensitivity of 84.2% (IC 95% 60.4–96.4) and specificity of 70.8% (IC 95% 55.9–83.0), VPP of 53.5, and VPN of 91.9.

When evaluating the risk of depression in patients with a curable prognosis, the cut-off point of the DT was ≥ 6, with AUC of 0.71 (IC 95% 0.6–0.8, *p* < 0.0262), sensitivity of 72.7% (IC 95% 39.1–93.7) and specificity of 77.6% (IC 95% 65.8–86.9), VPP of 34.8, and VPN of 94.5 (Table 4). In patients with an incurable prognosis, the cut-off point was of ≥ 4, with AUC of 0.8 (IC 95% 0.69–0.89, *p* < 0.0001), sensitivity of 83.3% (IC 95% 51.6–97.4) and specificity of 63.6% (IC 95% 49.6–72.7), VPP of 33.3, and VPN of 94.6.

In relation to the total scale (TADS-T), the cut-off point established for the DT in patients with a curable prognosis was > 4, with AUC of 0.83 (IC 95% 0.7–0.9, *p* < 0.0001), sensitivity of 69.2% (IC 95% 48.2–85.6) and specificity of 84.6% (IC 95% 71.9–93.1), VPP of 69.2, and VPN of 84.6. In patients with an incurable prognosis, the cut-off point was of ≥ 4, with AUC of 0.73 (IC 95% 0.6–0.8), sensitivity of 65.4% (IC 95% 44.3–82.8) and specificity of 68.3% (IC 95% 51.9–81.9), VPP of 56.7, and VPN of 75.7.

## Discussion

Ultra-brief screening instruments such as DT, although they do not have the ability on their own to diagnose depression or anxiety disorders, are useful to detect, at an early stage, the patients who are more prone to developing these disorders [22]. In Chile, there are no studies that evaluate screening methods for ED, which is why this study contributes to an advancement on this objective.

The DT has been used in several countries and languages, showing adequate psychometric properties [23, 24]. The results of our study are similar to those of the reported studies. In a recent systematic review of screening instruments for ED [25], the cut-off point to identify ED in a clinically relevant manner was of 4 or 5, depending on the validation measures used. The sensitivity and specificity were lower than 80% in half and the two-thirds of the validation studies, respectively. The higher levels of sensitivity contrasted with the moderate or low levels of specificity. Complementary studies suggest that modifications to the DT, such as the mood DT and the impact DT, may represent an advancement in relation to the original scale.

Our study reported an ED prevalence of 32.5% (DT ≥ 5), similar to what was detected in large sample studies [3, 4].

In spite of the prevalence of ED in oncology patients, there are few studies that have evaluated the research methods used by health-care professionals. Lawrie *et al*. [26] surveyed 134 physicians who worked in palliative care; 73% reported having routinely evaluated depression symptoms in their patients, of which only 50% used standardised instruments, 10% used one single question (‘are you depressed?’), and 27% used the HADS. Physicians show a tendency to underestimate depression symptoms when these are more severe and seem to be more influenced by symptoms such as crying and a depressive mood than by more specific symptoms such as anhedonia, suicidal thoughts, despair, and feelings of guilt [27].

In this respect, it shows the efficiency of the DT as an research tool for detecting ED in Chilean oncology patients who are about to begin chemotherapy, their best performance is in screening anxiety symptoms, with a cut-off point of ≥ 4, obtaining a sensitivity of 93.1% and a specificity of 87.8% in relation to HADS. The lower cut-off points for the DT do not provide any comparative advantages neither for anxiety nor for depression, decreasing considerably the specificity of the instrument.

The LP complements the results of the DT by determining which is the area of greater concern for the patient. In this study, the main problems noted by patients were of physical nature (88.0%), followed by emotional (80.1%) and practical (53.6%).

The advantage of this instrument lies in the possibility of a brief standardised evaluation that can be completed by the patient without support from the health personnel, providing a quick result that increases the detection threshold and facilitates timely referral without affecting the time needed for attention.

Our work has some limitations. The number of patients was relatively low, which is why the results are not easy to generalise. Different cancer types of oncology pathologies were considered, which does not allow for a more appropriate evaluation of the differences in ED related to the location or type of cancer. In our study, the most prevalent diagnoses, such as breast and colon cancer, did not show considerable differences with those of other locations, with regard to ED. The instruments were used the moment before the start of chemotherapy, which is why it is not possible to compare them with other moments during treatment. The prevalence of anxiety, depression, and ED was not considerably different in patients whose pathology is incurable versus curable. This is an interesting finding that will have to be analysed in greater detail in a future study. It is possible that the moment of measuring, before the start of chemotherapy, generates anxiety and fear at the same time, and provides the patient with the hope for partial or definitive improvement.

## Conclusion

It is possible to offer oncology patients with considerable ED the option for a timely referral and effective treatment. This is reinforced by the increase in publications that refer to a considerable reduction in anxiety and depression symptoms for high-risk patients who are given psycho-oncological interventions aimed at maintaining their autonomy, providing defence mechanisms, strengthening hope and confidence, and ensuring good communication with the health-care team [28]. These results extended to even a year after the intervention and corroborate the importance of timely screening of ED and support in the initial stages.

For the first time, a study has been carried out that evaluated DT in the Chilean population. The instrument shows a behaviour similar to that of its application in other populations, and is established as an efficient initial screening tool for Chilean patients.

## Figures and Tables

**Table 1.** Patient characteristics, type of cancer, and prognosis.

Characteristics	Total		Average	Range
*N*	166			
**Age**			54.2	16–79
**Gender**				
Women	109	(65.6%)		
Men	57	(34.5%)		
**Type of cancer**				
Breast	46	(27.9%)		
Head and neck	5	(3.0%)		
Colorectal	33	(19.9%)		
Lung	19	(11.4%)		
Gynaecological	10	(6.0%)		
Digestive	26	(15.7%)		
Urological	4	(2.4%)		
Hematologic	14	(8.4%)		
Other	9	(5.4%)		
**Prognosis**				
Curable	78	(47.3%)		
Incurable	67	(40.6%)		
Undetermined	21	(12.7%)		

**Table 2.** Prevalence of anxiety, depression, and ED symptoms and problems.

	Prevalence (%)		
	Total	By gender	
		W	M *p* value
**HADS**			
Anxiety (HADS-A)	32.7	37.0	25.0 0.12
Depression (HADS-D)	15.7	18.3	10.7 0.23
Total (HADS-T)	39.8	42.2	33.9 0.30
**DT**			
Distress (DT)	32.5	38.5	21.4 0.03
**LP**			
Practical problem	53.6	55.0	51.8 0.69
Family problem	22.3	24.8	17.9 0.31
Emotional problem	80.1	81.7	78.6 0.64
Spiritual problem	25.9	30.3	17.9 0.08
Physical problem	88.0	89.9	83.9 0.26

HADS: Hospital Anxiety and Depression Scale.

DT: distress thermometer.

LP: list of problems.

W: women.

M: men.

**Table 3.** Sensitivity and specificity of the DT with relation to HADS.

Screening	Cut-off point	Sensitivity		Specificity		VPP	VPN	AUC
		%	IC 95%	%	IC 95%			
Anxiety	≥ 4	88.9	77.4–95.8	78.4	69.6–85.6	66.7	93.5	0.89
Depression	≥ 5	69.2	48.2–85.6	74.3	66.2–81.3	33.3	92.9	0.76
Total	≥4	68.2	55.6–81.4	73.0	63.2–81.4	62.5	77.7	0.77

IC: trust intervals.

VPP: positive predictive value.

VPN: negative predictive value.

AUC: area under the curve.

**Table 4.** Sensitivity and specificity of the DT with relation to HADS, according to the prognosis.

Prognosis	Screening	Cut-off point	Sensitivity		Specificity		VPP	VPN	AUC
			%	IC 95%	%	IC 95%			
Curable	Anxiety	≥ 4	93.1	77.2–99.0	87.8	75.2–95.3	81.8	95.6	0.93
	Depression	≥ 6	72.7	39.1–93.7	77.6	65.8–86.9	34.8	94.5	0.71
	Total	≥ 5	69.2	48.2–85.6	84.6	71.9–93.1	69.2	84.6	0.83
Incurable	Anxiety	≥ 4	84.2	60.4–96.4	70.8	55.9–83.0	53.5	91.9	0.86
	Depression	≥ 4	83.3	51.6–97.4	63.6	49.6–76.2	33.3	94.6	0.80
	Total	≥ 4	65.4	44.3–82.8	68.3	51.9–81.9	56.7	75.7	0.73

IC: trust intervals.

VPP: positive predictive value.

VPN: negative predictive value.

AUC: area under the curve.
